# Intra-Subject Variability in Mathematical Learning Difficulties

**DOI:** 10.5334/joc.226

**Published:** 2022-05-27

**Authors:** Sharon Levy, Liat Goldfarb

**Affiliations:** 1 Edmond J. Safra Brain Research Center for the Study of Learning Disabilities, University of Haifa, IL

**Keywords:** mathematical learning difficulties, intra-subject variability, numerical cognition

## Abstract

**Objective:**

Mathematical learning difficulties (MLD) are characterized by difficulties in the understanding and processing of numbers and quantities. While MLD is related mainly to numerical deficits, studies show that this population has several other cognitive difficulties. The current study examined whether the cognitive deficits consisting of cognitive instability in the form of intra-subject variability (ISV) will also characterize the performance of individuals with MLD.

**Method:**

Female adults with MLD and a matched control group performed numerical and non-numerical tasks and various ISV measures were compared between the two groups.

**Results:**

Overall, the results showed that participants with MLD had higher ISV measures, including SD, sigma, and tau, only when performing numerical tasks.

**Conclusions:**

It appears that the cognitive system of MLD participants is less consistent and noisier when performing these numerical tasks. However, this inconsistency is not a general deficiency but rather a numerically specific one, as this inconsistency does not seem to characterize the performance of individuals with MLD in tasks that do not involve numerical processing. These findings have unique importance for understanding the difficulties characterizing individuals with MLD and possible future interventions.

## Introduction

### Mathematical learning difficulties and attention

Mathematical learning difficulties (MLD) relate to learning difficulties in the arithmetic domain. According to the literature, the estimated prevalence of MLD is similar to that of dyslexia and ADHD, and about 3–6% of school aged children demonstrate MLD. In addition, the numerical difficulties experienced by young individuals with MLD usually persist into adulthood (e.g., Shalev et al., 2000; Shalev et al., 2005).

There are several definitions of MLD. Some refer to MLD as developmental dyscalculia (DD) (e.g., Bartelet et al., 2014), while others suggest that DD and MLD reflect different disabilities (e.g., Rubinsten & Henik, 2009). The current paper does not aim to distinguish between these terms (for a review on DD see Kaufmann et al., 2020), but rather uses the term MLD for individuals who have significant difficulties in mathematical performance (e.g., Fritz et al., 2019; Furlong et al., 2015; Karagiannakis et al., 2014). These difficulties in the arithmetic domain can affect not only school performance but also everyday life (Butterworth, 2008). For example, daily activities such as calculating change or estimating the cost of grocery shopping can be difficult tasks when the basis of numerical understanding is deficient.

There are several theories and findings suggesting that MLD is also connected to general cognitive difficulties (that are not related to numbers). For example, adults with MLD were found to have difficulties in the attention alerting system, which is the system involved in the activation and preservation of attention for a specific stimulus (Askenazi & Henik, 2010). The alerting system is important for numerical skills since, for example, it has been suggested to facilitate the perception of small amounts (up to 4 items) in adult participants (Gliksman et al., 2016; Gliksman & Henik, 2019). The deficiencies in the alerting system among adults with MLD might be related to the IPS brain area, since the IPS has been found to be related to both numerical processing (Ansari et al., 2007; Butterworth et al., 2011; Rubinsten & Henik, 2009) and the alerting system (Fan et al., 2005; Rubinsten & Henik, 2009).

Attention difficulties in adults with MLD were also found in relation to inhibition. Adults with MLD showed a larger congruency effect compared to a control group, when performing a flanker task that required focusing on a center stimulus while inhibiting the response to flankers on both sides. It has been suggested that a deficit in inhibition or conflict resolution can be related to the difficulties MLD experience in different numerical tasks. For example, in arithmetic fact retrieval one must inhibit related but irrelevant numerical information in order to retrieve the correct solution. A deficit in inhibition might create difficulties in solving an expression correctly (Askenazi & Henik, 2010). A deficit in inhibiting irrelevant information is related not only to number fact retrieval but also to procedural skills (e.g., Abramovich & Goldfarb, 2015; Cragg et al., 2017).

Other studies have found general difficulties in inhibition (that are not specific to arithmetic) not only in adults but also in children with low mathematical abilities. For example, a correlation was found between mathematical ability and performance in the Wisconsin Card Sorting Task (WCST), which requires learning a sorting strategy and then changing this strategy and learning a new one (Bull & Scerif, 2001). While the WCST involves cognitive flexibility, it also requires inhibition of irrelevant old strategies in order to effectively switch to a new one (e.g., Buchsbaum et al., 2005). In Bull and Scerif (2001) the authors found a correlation between math ability and the inhibition of an established strategy in favor of a new and more efficient strategy. This type of inhibition (and shifting) is important for mathematics when learning new concepts and procedures, in order to inhibit a previous automatic procedural approach (Cragg & Gilmore, 2014). Other studies show that the implications of deficits in inhibition on math performance are different for children and adults. For example, while inhibition abilities were a predictor of procedural skills in children, they were related to conceptual understanding in adults (Gilmore et al., 2015).

While there are findings suggesting that individuals with MLD have general deficits in different aspects of attention and executive functions, there is also evidence showing that individuals with MLD have cognitive difficulties that are specific to the numerical environment, meaning that they are manifested while performing numerical tasks. For example, adults with MLD were found to present a larger alerting effect than controls with the presence of an alerting cue, when performing an enumeration task. In addition, while a control group showed an alerting effect for small amounts (up to 4) but not for larger amounts (5 and up), in the MLD group this effect was present for both ranges (Gliksman & Henik, 2019). These results support findings of a deficit in the alerting system in general among adults with MLD (Askenazi & Henik, 2010) and show that this deficit is also present in a task of numerical nature. As mentioned, it has been suggested that the alerting system facilitates the perception of small amounts (up to 4 items) in adult participants (Gliksman et al., 2016; Gliksman & Henik, 2019).

Another aspect of executive function found to be deficient in MLD when performing tasks of numerical nature is related to working memory (WM). Adults with math difficulties had more errors than controls in a task of serial order WM of digit list. It has been suggested that serial order WM is important for complex calculation, when maintenance of numerical information in the correct serial position is crucial for updating intermediate calculation results in order to reach a final correct solution (Attout et al., 2015). In general, working memory is important for various numerical skills such as single-digit and multi-digit arithmetic (Raghubar et al., 2010), and symbolic numerical comparison (Maloney et al., 2019).

Studies also show difficulties in inhibiting irrelevant information among individuals with MLD when performing arithmetic tasks. Adults with MLD showed difficulties associating Arabic numerals with their internal quantity representation as automatically as controls. Participants performed a physical Stroop task that required attention to the physical dimension of numerals while ignoring their numerical value. The MLD group showed a significantly smaller congruity effect compared to controls. However, participants with MLD were able to automatically associate letters with their phonemes (sounds) as well as controls (Rubinsten & Henik, 2006). It has been suggested that the lack of automaticity in processing numerical information causes individuals with MLD to invest more attentional resources compared to controls when processing numerical information (Ashkenazi et al., 2009).

Deficits in inhibition when performing numerical tasks were also found for children with mathematical difficulties. They were found to have difficulties with the inhibition of numerical irrelevant information associated with the task (e.g., solving 5+3 as 6, which is the number that follows the addend 5). However, the mathematical difficulties were not associated with a deficit in the activation of phonetic representations, as manifested in the articulation of familiar words. Inefficient inhibition specifically regarding numerical information is thought to be related to difficulties in arithmetic fact retrieval in individuals with MLD (Geary et al., 2000).

In another study, children with low math abilities were found to have lower accuracy levels compared to controls only in incongruent trials (and not in congruent trials) in a quantity comparison task, meaning that they had difficulties inhibiting information irrelevant to the numerical task (Wilkey et al., 2020). Inhibition in non-symbolic numeracy processing is important for the suppression of irrelevant non-numerical (visual) variables (e.g., Leibovich & Ansari, 2016). The relations between inhibition and numeracy might be different for children and adults due to factors such as the development of inhibitory control abilities (Szucs et al., 2013).

In summary, MLD can be associated with several cognitive deficits; some of them are general and some specific to the numerical field. The purpose of the current study is to investigate other cognitive difficulties among the MLD population, while examining the issue of generality versus numerical specificity. Specifically, the current study will examine cognitive deficiencies related to intra-subject variability among individuals with MLD.

### Intra-subject variability

Most of the studies that measure human cognitive performance investigate the level of accuracy and/or central measurements such as mean RT. However, human cognitive performance can also be measured by measurements related to within-subject variability or intra-subject variability (ISV). Cognitively, these measurements reflect performance inconsistency and rapid fluctuations in task performance. The ISV measures an important trait of the cognitive system – its consistency, such that high consistency of the cognitive system will be manifested in low ISV measures (e.g., Hultsch et al., 2002). When slow mean RT is found in a study, it usually leads to a conclusion related to the difficulty to perform a task in general. However, high ISV can occur when a participant performs a task at a normal speed in some trials and much slower or faster in others. Hence, high ISV is attributed to less consistent cognitive performance characterized by wide fluctuations.

Notably, while researchers frequently treat ISV as statistical noise or as a random error of the mean RT measurement, ISV is not noise – rather it is a real and consistent trait of one’s cognitive system. In fact, the degree of variability of a subject is highly predictable across tasks and time, as found for typically developing participants, and it is a real measurement of an important trait of the cognitive system – inconsistency (Hultsch et al., 2002).

The inconsistency of the cognitive system has been previously attributed to a less efficient general attention system (e.g., Ratcliff, 1979; Rosch et al., 2013; Tamnes et al., 2012). It has been attributed to the failure of the attention system to stay focused on the task. There have also been indications regarding different patterns of brain activation for attention focus in task-related activity compared to situations in which an individual is not actively engaged in a task. The first involves the “task-positive network” and the latter the “default mode network”. The two networks are negatively correlated, such that when the default mode network is activated the task-positive network is deactivated, and vice versa. Interestingly, participants with a stronger negative correlation between these two networks were also found to show more consistent behavioral performance as measured by ISV (Kelly et al., 2008). Increased ISV was also attributed to a less efficient cognitive system as it was related to failure in selecting and preparing the correct response in a task (e.g., Mostofsky & Simmonds, 2008), as well as failure in executive guidance for efficient processing (e.g., Rao et al., 2014).

ISV is also an important measurement since it can capture differences between special populations and control groups. Among these populations are individuals on the autistic spectrum (e.g., Geurts et al., 2008), those with mental disorders, including schizophrenia, depression, and borderline personality disorder (e.g., Kaiser et al., 2008), reading difficulties (e.g., Van De Voorde et al., 2010), and attention deficit/hyperactivity disorder (ADHD) (e.g., Geurts et al., 2008; Klein et al., 2006; Seernani et al., 2020; Van De Voorde et al., 2010).

For example, a study that compared a group of children with ADHD to a control group in their performance on attention tasks, found higher ISV among the ADHD group. Moreover, ISV measures were better measurements than mean RT and accuracy measures for distinguishing between the two groups (Klein et al., 2006). Similar results were found in a different study, where high ISV was found related not only to participants with ADHD but also to participants with reading difficulties (Van De Voorde et al., 2010).

There are different approaches to calculating intra-subject variability. One of the most commonly used measures is the intra-individual standard deviation (SD), which is calculated as the mean standard deviation of each individual across multiple RT trials (Lovden et al., 2007; Tamnes et al., 2012). This measure has several advantages, since it is very simple and easy to calculate and understand. However, the SD can be problematic as a measure of intra-subject variability since it can be related to the individual’s RT, such that increased mean RT is associated with increased SD. According to this notion, if two groups differ in their mean RT, statistically they are more likely to demonstrate a different SD, so SD is not the ideal measurement of ISV for comparing groups (Dykiert et al., 2012).

A possible solution to this problem is to apply another approach for analyzing the variability of performance using the ex-Gaussian distributional model. This model assumes that individual RT distribution has the components of both normal function and exponential function. The ex-Gaussian distribution consists of analysis of three parameters: mu, sigma, and tau. The mu (µ) reflects the mean of the normal function, sigma (σ) reflects the variation of the normal distribution, and tau (τ) represents the tail of the exponential distribution, which reflects the most deviant RTs. Taking all these three measurements into consideration enables a finer and more detailed understanding of how RT behaves in a certain task. It allows a more thorough examination of differences between groups, while taking into account both variability (as reflected by sigma) and extreme performance (as reflected by tau) in addition to the mean measure (mu) (Mcauley et al., 2006). The main shortcoming of this technique is that it is not suitable for most studies, as for optimal implementation it requires a relatively large amount of correct trials in each of the experimental conditions (Ratcliff, 1979).

#### The current study

As mentioned, studies show that the MLD population has several cognitive difficulties of both general and numerical nature. However, cognitive deficits related to inconsistency of the cognitive system, as manifested in ISV measures, have never before been thoroughly investigated in an MLD population.

The findings on high ISV among special populations, especially concerning learning difficulties, as well as the cognitive deficiencies observed in this population as reviewed above, raise the possibility that individuals with mathematical learning difficulties will also have high cognitive inconsistency compared to a control group.

The current study will be the first to thoroughly investigate the relationship between cognitive inconsistency in the form of ISV measures and mathematical learning difficulties. In addition, it will examine whether the inconsistency of the cognitive system, will characterize the performance of individuals with MLD in a variety of general tasks or only in tasks of a numerical nature. Specifically, participants with MLD and a matched control group will perform different types of tasks: numerical and non-numerical tasks, and ISV measures will be compared between the two groups. The study will examine whether individuals with MLD indeed have higher intra-subject variability and, if so, whether it is general or specific to the numerical domain. In order to do so, the study will include a comprehensive analysis encompassing different measures of ISV in addition to mean RT analysis.

If inconsistency of individuals with MLD is restricted to numerical tasks, it is hypothesized that only in numerical tasks (and not in non-numeric tasks) these individuals will show higher ISV measures compared to controls. Another possibility is that cognitive instability in individuals with MLD are not necessarily restricted to that area alone. According to this notion, participants with MLD will have higher ISV compared to controls in the numerical tasks but also in the non-numeric tasks.

## Method

### Participants

The research participants consisted of 16 female adults with MLD aged 23–33 (M = 28.31, SD = 3.18) and a matched control group of 16 adults with no learning difficulties or attention disorders aged 22–33 (M = 27.19, SD = 3.21). The two groups did not differ significantly in age [*t*(30) = .10, *p =* .33]. All participants signed an informed consent form and were paid for their participation (approximately $21–$35). All procedures in the study were approved by the Ethical Committee of the Faculty of Education, University of Haifa (approval number: 14/048), and the research was completed in accordance with Helsinki Declaration.

### Classification and assessment

All participants performed computerized numerical tests from the “Israeli learning function diagnosis system” (MATAL, 2007) for high school and higher education students. The MATAL was developed by the National Institute for Testing and Evaluation and is used to diagnose learning difficulties, including MLD, by conducting a large set of nationally normalized tests. Participants performed two numerical tests that were previously used to discriminate between MLD and controls (e.g., Furman & Rubinsten, 2012). The first consisted of simple calculation tasks (e.g., 2 + 2 = 4, 2*2 = 5, 8–4 = 4, 6:4 = 2) and the second of procedural knowledge calculation tasks (e.g., 750 + 10 = 760, 204–5 = 201, 20*20 = 400, 400:5 = 45). In both tasks, the participants were asked to report on the authenticity of the equation appearing on the screen, while accuracy and speed were measured by the computer.

In order to be classified for the MLD group, the average of all four measures described had to be below the 30^th^ percentile. In addition, in an interview performed by a learning difficulties specialist prior to the experiment, participants in the MLD group described major difficulties only in the arithmetic field, from the early years of elementary school through high school and until the current days. In order to be classified for the control group the average of all four measures described had to be above the 30^th^ percentile. In addition, all participants in this group stated that they had no learning difficulties or attention disorders.

In addition, all participants underwent a series of background academic tests to assess abilities, including: reading, non-verbal reasoning, verbal retrieval, attention, and basic arithmetic. Assessment of reading abilities was conducted using the One-minute test for words and the One-minute test for pseudo-words (Shatil, 1997a; Shatil, 1997b), in which participants were asked to read aloud as many correct words or pseudo-words as possible in one minute. Non-verbal reasoning was assessed using the Raven Progressive Matrices (Raven, 1960). A semantic fluency test and a phonological fluency test (Kave, 2006) were used to assess verbal retrieval abilities. Participants were asked to say as many words as possible in a specific category in one minute (for the semantic fluency test), and to say as many words as possible that begin with a specific letter in one minute (for the phonological fluency test). For basic arithmetic abilities participants underwent a 2-minute calculation test that consists of one-digit exercises in addition, subtraction, multiplication, and division (Openhaim–Biton & Breznitz, 2004). The participants were asked to solve correctly as many exercises as possible in two minutes. Assessment of attention was performed through an attention questionnaire based on the Diagnostic and Statistical Manual of Mental Disorders (American Psychiatric Association, 2013). The attention questionnaire was composed of two parts: (A) inattention items and (B) hyperactivity/impulsivity items, and was used as a self-assessment tool. The order of the tests was counterbalanced between participants with the following exceptions. In the reading tests, the One-minute test for pseudo-words always followed the One-minute test for words, in the retrieval tests the phonological fluency test always followed the semantic fluency test, and the attention questionnaire was always last (to avoid a priming effect).

Independent t-tests revealed no significant difference between the two groups in all of the non-numeric measures (reading words, reading pseudo-words, Raven Progressive Matrices, semantic fluency, phonological fluency, and attention) (all *p*s > .09). In the 2-minute calculation test, as expected, the MLD group performed significantly worse (M = 42.94, SD = 6.73) than the control group (M = 71.56, SD = 10.93) [*t*(24.94) = 8.92, *p* < .001] (see Table 1).

**Table 1:** Mean and SD in the assessment tests for each group, independent t-test scores, and p values.


		MLD	CONTROL	T-VALUE	P VALUE

**Word per min**		96.69 (11.38)	92.31 (22.49)	.69	.49

**Pseudo-words per min**		41.38(15.98)	50.31 (12.17)	1.78	.09

**Semantic fluency**		22.50 (5.38)	24.63 (6.62)	.10	.33

**Phonological fluency**		12.38 (3.36)	13.75 (5.22)	.89	.38

**Raven Progressive Matrices (raw scores)**		49.69 (5.02)	52.38 (3.59)	1.74	.09

**Attention questionnaire (sum of symptoms marked as “yes”)**	**Part A**	1.44 (1.75)	1.56 (1.93)	.19	.85

	**Part B**	2.13(1.59)	1.75(1.77)	.63	.53

	**Parts A+B**	3.56 (3.20)	3.31 (3.03)	.23	.82

**2 minute calculation test**		42.94 (6.73)	71.56 (10.93)	8.92***	<.001


### The experimental tasks

Four computerized tasks were conducted for each participant. The four tasks included two numerical tasks: numerical comparison and equations judgment, and two non-numerical tasks as a control tasks for each numerical task: shape comparison and semantic judgment (respectively).

#### Numerical comparison

*Stimuli*: This task was constructed according to Rousselle and Noël (2007). Each trial was composed of two digits from 1-9, presented on both sides of the screen. As in Rousselle and Noël (2007), the specific pairs were: 1–2, 2–3, 3–4, 1–4, 1–5, 2–5, 6–7, 7–8, 8–9, 5–8, 5–9, and 6–9. Each pair appeared both with the larger number on the right and the larger number on the left, creating 24 different trials (12 × 2). Each digit was written in font Arial, size 48, bold, with a distance of 7 cm between the two digits.

*Procedure*: Each trial began with a white interval that appeared for 500 ms, followed by the stimuli that remained on the screen until the participant responded. All stimuli appeared in black on a white background. The interval was a white background screen without a fixation cross, which might be interpreted as a plus signal (or multiplication signal) and might have created an interference with the numerical tasks. This was also the original design in Rousselle and Noël (2007) and in other numerical tasks such as those in the MATAL. Since two of the tasks in the current study were of numerical nature and the other two were their control tasks, the design of using a white interval was kept constant across all the other tasks.

Participants were instructed to report which digit has a larger numerical value by pressing one of two optional keys for either right or left. Each numerical comparison appeared 16 times, creating a total of 384 trials presented randomly. The task took about 7 minutes.

#### Shape comparison

*Stimuli*: This task was created as a control task for the numerical comparison. Each trial was composed of two shapes, X and O, presented on both sides of the screen. In half the trials the X appeared on the right side of the screen and in half on the left. Each shape was written in font Arial, size 48, bold, with a distance of 7 cm between the two shapes.

*Procedure*: Each trial began with a white interval that appeared for 500 ms, followed by the stimuli that remained on the screen until the participant responded. All stimuli appeared in black on a white background. Participants were instructed to report on the location of the X by pressing one of two optional keys for either right or left. As in the numerical comparison task, the shape comparison task also included a total of 384 trials presented randomly. The task took about 7 minutes.

#### Equations judgment

*Stimuli*: This task included two-digit addition equations, each containing two addends with either the correct or incorrect sum. Equations were constructed according to previous literature (Klein et al., 2010) with the following rules. The sum in all equations was between 61 and 99. Multiples of 10 (70, 80, 90) and ties (e.g., 22, 33) were not included either as addends or as sums. The unit digits and the tens digits of the two addends were never the same (e.g., 53 + 23, 41 + 48) and the two addends were not part of the multiplication table (e.g., 27 + 36). The task included 48 correct non-carry equations, of which each appeared once with the large addend first and once with the large addend second, creating a total of 96 non-carry equations. The incorrect equations were created according to the same rules mentioned above, with the sum changed in one of four options: ±2 or ±10. This was done in order to prevent solving of the equation based on one digit only (units or tens) or based on knowledge about the sum being even or odd. There were 6 incorrect non-carry equations of each type of error in the sum (+2, –2, +10, –10) creating a total of 24 different equations of this type. No correct equation appeared as an incorrect one as well.

In order to prevent participants from reporting on the authenticity of the equation by using strategies instead of solving it, filler equations that required carry were also included in the task. These equations were composed of two addends whose unit digits exceeded 10 when added (e.g., 34 + 28). All guidelines for creating the non-carry equations were also applied for creating the carry equations. Ten different correct equations and two incorrect equations were created as fillers. All equations were written in font Arial, size 26, bold.

*Procedure*: Each trial began with a white interval that appeared for 500 ms, followed by the stimuli that remained on the screen until the participant responded. All stimuli appeared in black on a white background. The equations appeared in the center of the screen, one at a time. Participants were asked to solve each equation and then report on its authenticity by pressing one of two optional keys for either right or wrong. In total, this task included 132 equations of four types: correct non-carry, incorrect non-carry, correct carry, incorrect carry. Incorrect equations comprised about 20% of the total number of equations (26/132). The task was divided into two blocks, each containing 66 equations chosen randomly by the computer from the total 132 equations. This was created to reduce tiredness, so that participants could rest and continue the task when they are ready while staying in the experimental room. The task took about 15 minutes.

#### Semantic judgment

*Stimuli*: This task was created as a control task for the equation judgment. Sentences written in Hebrew were modified from a study by Prior and Bentin (2006). In all sentences a noun appeared first, then a verb (with a proper proposition if necessary), and finally a second noun (e.g., “השחקנית מעשנת סיגריהיה”, the actress (first noun) is smoking (verb) a cigarette (second noun)). Half the sentences were composed of a total of three words and half had a total of four words (with a proposition). In half the sentences the verb was active and in half passive. As in the equations judgment task, this task contained 96 correct sentences, of which each appeared once. Twenty four incorrect sentences were created based on the correct sentences, changing either the first or the last noun such that the sentence did not make sense. Half the incorrect sentences consisted of a semantical violation in the first noun (e.g., “החולצה אפתה עוגה”, “the shirt baked a cake”) and half consisted of a semantical violation in the second noun (e.g., “הדגים שוחים בדשא”, “the fish swim in the grass”). As in the correct sentences, the incorrect sentences consisted of either three (50%) or four (50%) words and contained either active (50%) or passive (50%) verbs.

Since there was no verbal equivalent to the carry condition in the equations judgment, 10 additional correct sentences and two incorrect ones were included in the task as fillers. These sentences were based on the original sentences, with a change in either the first or last noun. The proportion of number of words (3 or 4) and the type of verb in the sentence (active or passive) was the same as in the original sentences. As in the equations judgment task, all sentences were written in font Arial, size 26, bold.

*Procedure*: Each trial began with a white interval that appeared for 500 ms, followed by the stimuli that remained on the screen until the participant responded. All stimuli appeared in black on a white background. Sentences appeared in the center of the screen one at a time. Participants were asked to read each sentence and then report on its semantical authenticity by pressing one of two optional keys for either right or wrong. As in the equation judgment task, this task included a total of 132 sentences of four types: correct sentences, incorrect sentences, correct sentences as fillers, and incorrect sentences as fillers. Incorrect sentences comprised about 20% of the total number of sentences (26/132). The task was divided into two blocks, each containing 66 sentences chosen randomly by the computer from the total 132 sentences. This was created to reduce tiredness, so that participants could rest and continue the task when they are ready while staying in the experimental room. The task took about 15 minutes.

#### General procedure

Each task began with a short practice block that contained five stimuli presented randomly. Then an experimental block began. Participants were asked to report their answer to each stimulus as quickly and accurately as possible. Response time and accuracy were measured by the computer.

The order of the tasks was counterbalanced so that each numeric task could be either before or after its non-numeric task and each of the two pairs of tasks (numeric task with its non-numeric task) could be either first or second. This created a total of four different orders of the tasks.

Presentation of stimuli and collection of data were administered using an HP Compaq computer with a 22-inch Samsung monitor. The experimental tasks were programmed and presented using E-Prime 2.0. The participants sat at a distance of about 60 cm from the screen and a keyboard was placed next to it. Three participants from each group performed the experiment on a Dell Latitude E5530 laptop with a 15.6-inch screen. The resolution of all experiments was 1024X768.

## Results

Trials in which participants did not answer correctly were not included in the analysis (between 1–3% on average for each task). All participants performed above 90% accuracy in each of the tasks. The mean RT/SD for each participant in each task was calculated.

### Mean RT analysis

**Figure 1:**
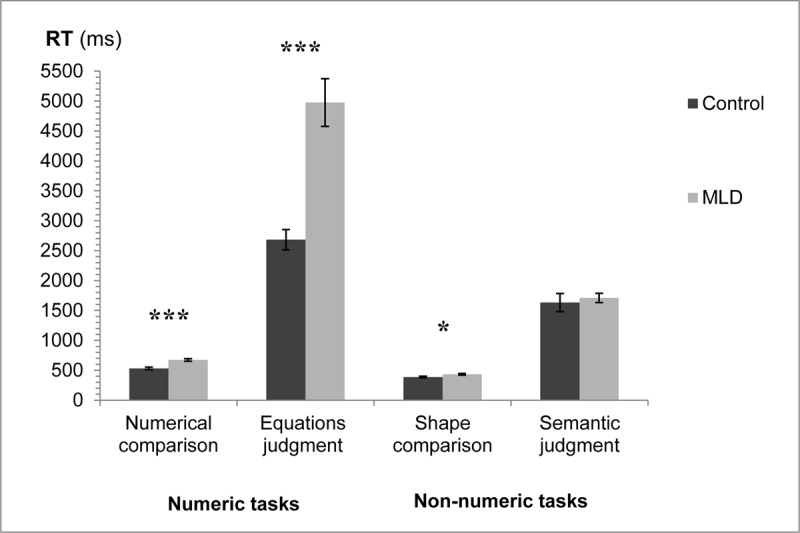
RT for the two groups in the different tasks. Error bars represent SE. * *p* < .05 *** *p* < .001.

A three-way analysis of variance was applied to the mean RT with numeric/non-numeric and task (comparison/judgment) as within participant factors, and group (MLD/control) as a between participant factor. See Figure 1 for mean RTs in the different conditions.

The interaction of interest between group and numeric/non-numeric was significant, *F*(1,30) = 29.26, *MS_e_* = 365,113, *p* < .001, η_p_^2^ = 0.49. There was also a significant interaction between all three factors *F*(1,30) = 26.43, *MS_e_* = 340,030, *p* < .001, η_p_^2^ = 0.47. In addition, significant main effects were found for all three factors: group (MLD/control), numeric/non-numeric, and task (comparison/judgment). There was a significant interaction between group and task, as well as between numeric/non-numeric and task. All *ps* < .001.

Further analysis of the interactions between the three factors revealed that in the comparison condition there was a significant interaction between group and numeric/non-numeric, *F*(1,30) = 21.59, *MS_e_* = 1695.18, *p* < .001, η_p_^2^ = 0.42. This suggests that the difference in mean RT between MLD and controls was greater for the numeric comparison task than for the non-numeric shape comparison task. The MLD group had significantly slower mean RTs compared to the control group in the numeric task (numerical comparison), *F*(1,30) = 20.87, *MS_e_* = 7750, *p* < .001, η_p_^2^ = 0.41 as well as in the non-numeric task (shape comparison), *F*(1,30) = 5.79, *MS_e_* = 2995.45, *p* < .05, η_p_^2^ = 0.16. In the judgment condition there was also a significant interaction between group and numeric/non-numeric, *F*(1,30) = 27.91, *MS_e_* = 703,449, *p* < .001, η_p_^2^ = 0.48. This suggests that the difference in mean RT between MLD and controls was greater for the numeric equations judgment task than for the non-numeric semantic judgment task. The MLD group had significantly slower mean RTs compared to the control group in the numeric task (equations judgment), *F*(1,30) = 28.07, *MS_e_* = 1,500,161, *p* < .001, η_p_^2^ = 0.48. However, no significant difference was found between the two groups in the non-numeric task (semantic judgment), *F* < 1 (Figure 1).^1^

### SD analysis

**Figure 2:**
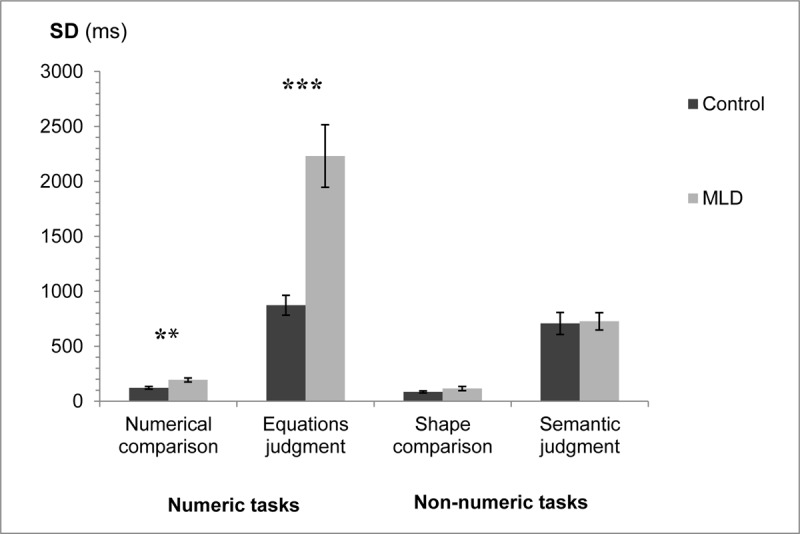
SD for the two groups in the different tasks. Error bars represent SE. ** *p* < .01 *** *p* < .001.

A similar analysis was applied to the SD measurement. A three-way analysis of variance was applied to the mean SD of each participant, with numeric/non-numeric and task (comparison/judgment) as within participant factors, and group (MLD/control) as a between participant factor. See Figure 2 for mean SDs in the different conditions.

The Interaction of interest between group and numeric/non-numeric was significant, *F*(1,30) = 24.51, *MS_e_* = 155,457, *p* < .001, η_p_^2^ = 0.45. There was also a significant interaction between all three factors, *F*(1,30) = 20.83, *MS_e_* = 161,810, *p* < .001, η_p_^2^ = 0.41. In addition, significant main effects were found for all three factors: Group (MLD/control), numeric/non-numeric, and task (comparison/judgment). There was a significant interaction between group and task, and also between numeric/non-numeric and task. All *p*s *<* .001.

Further analysis of the interactions between the three factors revealed that in the comparison tasks the interaction between group and numeric/non-numeric was marginally significant, *F*(1,30) = 4.0, *MS_e_* = 1688.76, *p* < .055, η_p_^2^ = 0.12. The MLD group had significantly higher SDs compared to the control group in the numeric task (numerical comparison), *F*(1,30) = 11.08, *MS_e_* = 3666.2, *p* < .01, η_p_^2^ = 0.27. No significant difference was found between the two groups for the non-numeric task (shape comparison), *F*(1,30) = 1.86, *MS_e_* = 3925.73, *p =* .18, η_p_^2^ = 0.06. In the judgment tasks there was a significant interaction between group and numeric/non-numeric, *F*(1,30) = 22.74, *MS_e_* = 315,578, *p* < .001, η_p_^2^ = 0.43. The MLD group had significantly higher SDs compared to the control group in the numeric task (equations judgment), *F*(1,30) = 20.65, *MS_e_* = 715,321, *p* < .001, η_p_^2^ = 0.41. However, no significant difference was found between the two groups for the non-numeric task (semantic judgment), *F* < 1 (Figure 2).^2^

### Ex-Gaussian analysis

In order to examine more thoroughly the ISV differences between MLD and controls, ex-Gaussian analyses were conducted for all three measures: mu, sigma, and tau.

### Mu analysis

**Figure 3:**
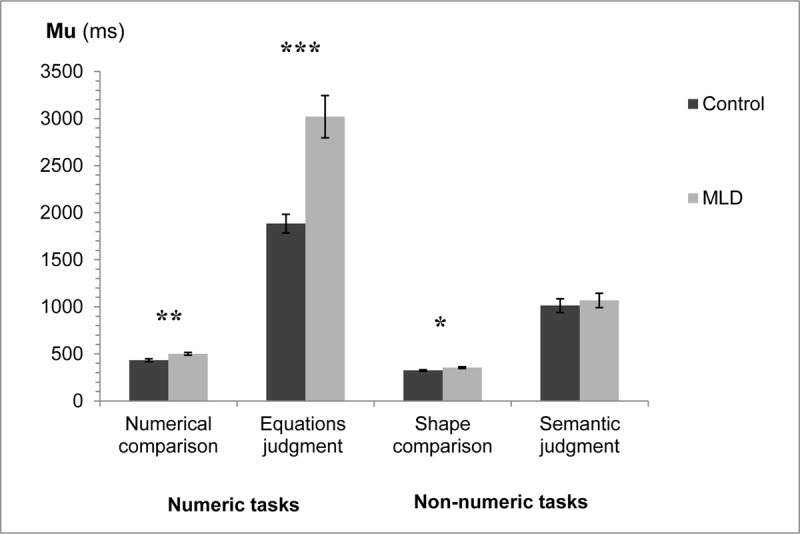
Mu for the two groups in the different tasks. Error bars represent SE. * *p* < .05 ** *p* < .01 *** *p* < .001.

A three-way analysis of variance was applied to the mean mu with numeric/non-numeric and task (comparison/judgment) as within participant factors, and group (MLD/control) as a between participant factor. See Figure 3 for mean mu’s in the different conditions.

Interaction of interest between group and numeric/non-numeric was significant, *F*(1,30) = 19.25, *MS_e_* = 130,268, *p* < .001, η_p_^2^ = 0.39. There was also a significant interaction between all three factors, *F*(1,30) = 16.51, *MS_e_* = 132,054, *p* < .001, η_p_^2^ = 0.35. In addition, significant main effects were found for all three factors: group (MLD/control), numeric/non-numeric, and task (comparison/judgment). There was a significant interaction between group and task, as well as between numeric/non-numeric and task. All *ps* < .001.

Further analysis of the interactions between the three factors revealed that in the comparison condition there was a significant interaction between group and numeric/non-numeric, *F*(1,30) = 7.67, *MS_e_* = 750.35, *p* < .01, η_p_^2^ = 0.20. This suggests that the difference in mean mu between MLD and controls was greater for the numeric comparison task than for the non-numeric shape task. The MLD group had significantly higher mu’s compared to the control group in the numeric task (numerical comparison), *F*(1,30) = 11.09, *MS_e_* = 3400.47, *p* < .01, η_p_^2^ = 0.27. This difference was also found in the non-numeric task (shape comparison), *F*(1,30) = 6.77, *MS_e_* = 1113.78, *p* < .05, η_p_^2^ = 0.18. In the judgment condition as well there was a significant interaction between group and numeric/non-numeric, *F*(1,30) = 17.90, *MS_e_* = 261,572, *p* < .001, η_p_^2^ = 0.37. Again, the MLD group had significantly higher mu’s compared to the control group in the numeric task (equations judgment), *F*(1,30) = 21.30, *MS_e_* = 485,139, *p* < .001, η_p_^2^ = 0.42. However, no significant difference was found between the two groups in the non-numeric task (semantic judgment), *F* < 1. (Figure 3).^3^

### Sigma analysis

**Figure 4:**
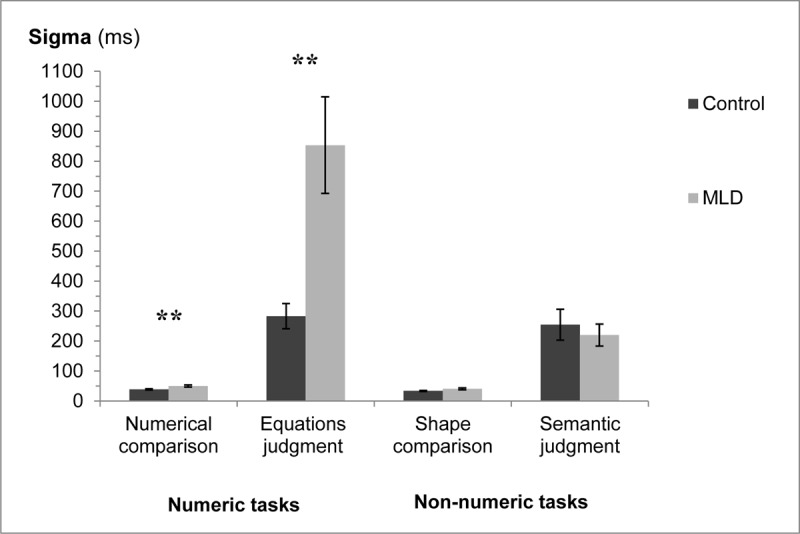
Sigma for the two groups in the different tasks. Error bars represent SE. ** *p* < .01.

A three-way analysis of variance was applied to the sigma data, with numeric/non-numeric and task (comparison/judgment) as within participant factors and group (MLD/control) as a between participant factor. See Figure 4 for the different sigma value in the different conditions.

The interaction of interest between group and numeric/non-numeric was significant, *F*(1,30) = 15.42, *MS_e_* = 48,134, *p* < .001, η_p_^2^ = 0.34. There was also a significant interaction between all three factors, *F*(1,30) = 15.41, *MS_e_* = 46,860, *p* < .001, η_p_^2^ = 0.34. In addition, significant main effects were found for all three factors: group (MLD/control), numeric/non-numeric, and task (comparison/judgment). There was a significant interaction between group and task, as well as between numeric/non-numeric and task. All *p*s *<* .014.

Further analysis of the interactions between the three factors revealed that in the comparison condition there was no significant interaction between group and numeric/non-numeric, *F*(1,30) = 1.14, *MS_e_* = 60.25, *p =* .29, η_p_^2^ = 0.04. However, examination of the differences between the two groups in each type of task separately, revealed that the MLD group had significantly higher sigma’s compared to the control group in the numeric task (numerical comparison), *F*(1,30) = 7.74, *MS_e_* = 127.79, *p* < .01, η_p_^2^ = 0.21. This difference was not found in the non-numeric task (shape comparison), *F*(1,30) = 3.65, *MS_e_* = 106.43, *p =* .07, η_p_^2^ = 0.11. In the judgment condition there was a significant interaction between group and numeric/non-numeric, *F*(1,30) = 15.43, *MS_e_* = 94,934, *p* < .001, η_p_^2^ = 0.34. Again, the MLD group had significantly higher sigma’s compared to the control group in the numeric task (equations judgment), *F*(1,30) = 11.73, *MS_e_* = 222,099, *p* < .01, η_p_^2^ = 0.28. However, no significant difference was found between the two groups in the non-numeric task (semantic judgment), *F* < 1 (Figure 4).^4^

### Tau analysis

**Figure 5:**
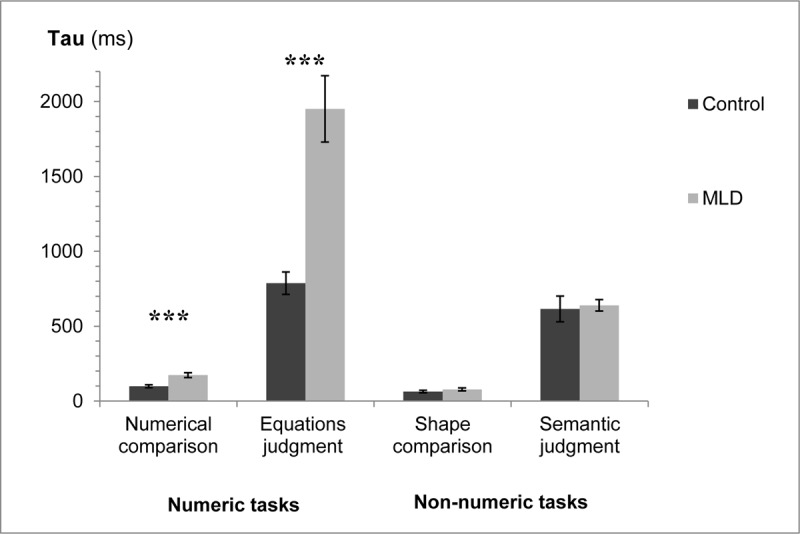
Tau for the two groups in the different tasks. Error bars represent SE. *** *p* < .001.

A three-way analysis of variance was applied to the tau data with numeric/non-numeric and task (comparison/judgment) as within participant factors, and group (MLD/control) as a between participant factor. See Figure 5 for tau data in the different conditions.

The interaction of interest between group and numeric/non-numeric was significant, *F*(1,30) = 28.15, *MS_e_* = 102,215, *p* < .001, η_p_^2^ = 0.48. There was also a significant interaction between all three factors, *F*(1,30) = 24.70, *MS_e_* = 94,330, *p* < .001, η_p_^2^ = 0.45. In addition, significant main effects were found for all three factors: group (MLD/control), numeric/non-numeric, and task (comparison/judgment). There was a significant interaction between group and task, as well as between numeric/non-numeric and task. All *p*s *<* .001.

Further analysis of the interactions between the three factors revealed that in the comparison condition there was a significant interaction between group and numeric/non-numeric, *F*(1,30) = 14.24, *MS_e_* = 1014.19, *p* < .001, η_p_^2^ = 0.32. The MLD group had significantly higher tau’s compared to the control group in the numeric task (numerical comparison), *F*(1,30) = 15.67, *MS_e_* = 2827.84, *p* < .001, η_p_^2^ = 0.34. This difference was not found in the non-numeric task (shape comparison), *F*(1,30) = 1.21, *MS_e_* = 1361.48, *p =* .28, η_p_^2^ = 0.04. In the judgment condition as well there was a significant interaction between group and numeric/non-numeric, *F*(1,30) = 26.56, *MS_e_* = 195,531, *p* < .001, η_p_^2^ = 0.47. Again, the MLD group had significantly higher tau’s compared to the control group in the numeric task (equations judgment), *F*(1,30) = 24.80, *MS_e_*=436,847, *p* < .001, η_p_^2^ = 0.45. However, no significant difference was found between the two groups in the non-numeric task (semantic judgment), *F* < 1 (Figure 5).^5^

## Discussion

The current study examined mathematical learning difficulties from a new perspective, addressing intra-subject variability in this population. The concept of fluctuations in response times relates to the notion that performing the same task by the same person can sometimes be slow and other times fast. These fluctuations in RT are attributed to a failure of the cognitive system to remain stable. They are neither statistical noise nor random errors – rather a real and consistent trait of one’s cognitive system. In fact, one’s degree of variability or inconsistency is highly predictable across tasks and time, as found for typically developing participants (e.g., Fuentes et al., 2001; Hultsch et al., 2002; Rabbitt et al., 2001). Hence, the current study investigated whether the cognitive instability related to high ISV measures characterize the performance of MLD and if so, whether they characterize a variety of general tasks or only tasks of a numerical nature. Adult participants with MLD and a matched control group performed numerical and non-numerical tasks, and various ISV measures (in addition to mean RT) were compared between the two groups. Results were analyzed using both orthodox and Bayesian statistics, and included examination of SD and ex-Gaussian measures in addition to the traditional measurement of mean RT.

Overall, the results showed more differences between MLD and controls in their performance of numeric tasks than in tasks that do not require numerical abilities. As in other studies, these differences were manifested when we examined central measurements related to mean RT. That is, these differences were manifested in slower mean RTs in the MLD group compared to the control group when performing numeric tasks than when performing non-numerical tasks. Similarly, when analyzing the mu measure, MLD were slower than controls when performing numeric tasks than when performing non-numerical tasks. More interestingly, the novelty of the current study is that MLD also showed higher ISV than controls only when performing numeric tasks. This was found when the SD measure was examined as well as when the ex-Gaussian measures representing the ISV were examined (sigma and tau). That is, MLD had higher SD, sigma, and tau, than the control group only when performing numerical tasks. Taken together, the results suggest that MLD are not only slower in performing numeric tasks in comparison to controls, but their cognitive system is less consistent and noisier when performing this type of task. However, this inefficiency does not seem to characterize MLD performance on tasks that do not involve numerical processing.

Although MLD has mathematical deficits (e.g., Fritz et al., 2019; Furlong et al., 2015; Karagiannakis et al., 2014; Rubinsten & Henik, 2009), the present study strengthens findings regarding additional cognitive difficulties in this population. As mentioned before, some studies demonstrate general cognitive deficits among MLD and DD (e.g., Askenazi & Henik, 2010; Bull & Scerif, 2001; Gliksman & Henik, 2019; Rubinsten & Henik, 2006; Wilkey et al., 2020). The current study is the first to systematically investigate another cognitive deficit among MLD, in the form of ISV.

The ISV measurements have unique importance since they reflect the consistency of the cognitive system. High ISV is thought to represent an inconsistency of the cognitive system (e.g., Hultsch et al., 2002), and is attributed to less efficient cognitive performance, characterized by wide fluctuations. This means that high ISV can occur when some trials of a specific task are performed at a normal pace and others at a much slower or faster pace. Possible approaches for analyzing the variability of performance and the variety of RTs in a specific task are the SD as well the ex-Gaussian distribution. In the present study MLD differ from controls in the numeric tasks in all three parameters of ISV: SD, as well as the ex-Gaussian measures of sigma and tau. While the sigma measurement represents variability in performance (Mcauley et al., 2006), the tau measurement reflects the most deviant RTs. This means that the tau represents extreme performance – the trials in which RTs are the slowest. The high tau among the MLD group in the numeric tasks suggests that their unusual slow trials were extremely slow compared to controls and their higher sigma compared to controls in the numerical task represent general higher variability. The high ISV measurements among the MLD group compared to controls suggests that when performing tasks of a numeric nature they have difficulties preforming the cognitive process necessary for stable performance.

Several notions might explain the inconsistency of the cognitive system in individuals with MLD, specifically when performing numerical tasks. For example, it has been suggested that MLD is related to lack of automaticity in processing numerical information (e.g., Ashkenazi et al., 2009). Less robust and less automatic numerical information might lead to less fluent processing, which might be related to greater inconsistency. Similarly, a deficiency in problem solving and decision making across multiple domains of mathematics is related to MLD (Menon et al., 2020). Hence, it is possible that individuals with MLD use a large variety of inefficient strategies in relation to numerical information, and this could lead to large inconsistency. This can also fit the notion of specific attentional deficiency when processing numerical information.

It has been suggested that RT fluctuations are related to a deficit in the attention system so that attentional lapses are reflected in a large variation in RT (e.g., Tamm et al., 2012). Similarly, large variations in RT were attributed to the failure of the attention system to focus on task-related features and to be actively engaged in a task (e.g., Kelly et al., 2008; Panagiotaropoulou et al., 2019). Hence, it is possible that MLD have a deficiency in retaining attention focused and engaged on the task when dealing with numerical information, and these lapses might result in a larger ISV.

This potential failure of the attention system can be another form of attention deficiency in MLD. As mentioned, individuals with MLD or DD were found to have difficulties in attentional and executive functions that are related to the numerical domain (Ashkenazi et al., 2009; Attout et al., 2015; Geary et al., 2000; Gliksman & Henik, 2019; Wilkey et al., 2020). In addition, it is worth noting that, as suggested before, attentional and executive processing are important for numerical processing. Working memory was found to be important for various numerical skills, such as arithmetic procedures (Raghubar et al., 2010) and symbolic numerical comparison (Maloney et al., 2019). Selective attention in the form of inhibition skills was found to be related to the basis of mathematics in adults (Gilmore et al., 2015) as well as to various numerical skills such as non-symbolic numeracy processing (e.g., Leibovich & Ansari, 2016), fact retrieval, and procedural skills (Cragg et al., 2017).

Although cognitive deficiencies in the form of high ISV measures were studied extensively previously in other special populations such as ADHD, they were never comprehensively studied in the context of MLD as suggested in the introduction. Since a large body of literature has studied cognitive instability in different populations such as ADHD, it seems important to connect MLD literature to this body of literature. This can help motivate new theoretical perspectives about numerical cognition and MLD, and clarify the nature and symptoms of MLD.

However this number-specific cognitive deficiency observed in MLD in the form of inconsistency is unique in the sense that in other populations such as the population with ADHD, the higher ISV measures are found in a variety of tasks (e.g., Geurts et al., 2008; Klein et al., 2006; Seernani et al., 2020; Van De Voorde et al., 2010). Interestingly, while the instability of the cognitive system seems to characterize ADHD in various situations, in the case of MLD this deficit is limited to the numerical domain.

While the main and important finding of this study is the differences between MLD and controls in their ISV measures while performing numeric tasks, it is also worth noting the finding regarding the difference found between the two groups in the central measures of mean RT and mu in the shape comparison task. While this task does not involve any numerical processing, it does require spatial reference of left and right. Previous findings point to spatial difficulties among the MLD population, in addition to the core numerical deficit (e.g., Ashkenazi et al., 2013; Rotzer et al., 2009; Szucs et al., 2013). The findings regarding spatial deficits among the MLD population can explain the difference found in the current study between the two groups when performing the shape comparison task. However, it is important to note that this difference was found only for mean measures of RT and mu, and not for any of the ISV measures. This strengthens the importance of addressing ISV measures in addition to mean measures when studying the difficulties characterizing individuals with MLD, since it seems that it is actually specific to the numerical context. When examining the performance of individuals with MLD using mean measures, their difficulties are found for a variety of tasks that are not necessarily numerical in nature. However, the inconsistency as manifested by high ISV measures seems to characterize their performance only in the numerical context.

The present study has some limitations worth addressing. The equation judgement task and the semantic judgment task differed in their mean RT in the control group. Hence, this can potentially reflect a certain limitation of the current study since the semantic judgment task is treated as a control task for the equation judgment task. On the other hand, all the intra variability measures, which were the main interest of the current study, were similar between the tasks in the control group. Meaning that when examining all three ISV measures (SD, sigma and tau) in the control group, their performance was similar between the equation judgement task and the control task: the semantic judgement task (as well as between the numerical and the control shape comparison tasks). In addition, the MLD group also showed similar performance to the control group in the non-numerical tasks (semantic judgment task and shape comparison task) in relation to all three ISV measures. Since the focus of the current study is the ISV measures (and not the mean RT measures), the semantic judgment task can in that sense be a control task for the equation judgement task. Another issue worth addressing is the use of a general self-reporting tool for evaluation of attention in both groups. This tool was based on the DSM V, and it is commonly used for diagnosis of ADHD. Since it was previously found that ADHD is related to cognitive inconsistency, this common tool was suitable for showing that there were no differences between the two groups in this aspect. However, other RT measures of specific attention functions, such as executive attention, were not specifically assessed in the current study and could limit the current conclusions.

The current study focused on examining ISV measures in individuals with MLD in numerical vs. non-numerical tasks. There were several findings regarding difficulties of children and adults with low math abilities in non-numerical tasks such as visuo-spatial working memory, visuo-spatial short-term memory, the trail making task, and Stroop tasks of conceptual size (Gliksman & Henik, 2018; Szucs et al., 2013; Szucs et al., 2014). A difficulty with a non-numeric task among MLD was also found in the current study for the shape comparison task. However, this difficulty was only manifested in slow RT compared to controls and not in high ISV measures as in the numerical tasks. Future studies can examine ISV measures in other non-numerical tasks in which individuals with MLD were found to be deficient.

Finally, it is worth noting that the current cognitive difficulties characterizing performance in tasks that require numerical processing might have implications for efficient interventions for the population with MLD, and can help to identify the specific conditions in which the cognitive deficiencies arise. If individuals with MLD indeed suffer from cognitive instability, considering this aspect when trying to maximize their numerical performance can help develop strategies and tools that focus specifically on these instability components. For example, if individuals with MLD indeed suffer from this inconsistency deficiency, long mathematical exercise sessions should be more thoroughly studied and might be reconsidered when dealing with MLD populations, as this practice can be connected to an increased level of cognitive instability (e.g., Walker & Trick, 2018). In fact, ISV measures provide new information on MLD that can allow better understanding of the difficulties associated with it and suggest appropriate future interventions.

## Data Accessibility Statement

Data of the study will be available at Figshare: 10.6084/m9.figshare.17025440

## Footnotes

Bayesian statistical analyses were also conducted in order to provide further support for the data, using JASP statistical software 0.9.2 with the default prior values (JASP, 2019; Rouder et al., 2012). Analyzing the significant difference between the two groups in their performance of the numeric comparison task revealed a BF_10_ of 259.65. This suggests that given the data, the experimental hypothesis (MLD will be slower than controls in performing the numeric comparison task) is 259.65 times more likely than the alternative. The same pattern of results was found for the equations judgment task, and the analysis revealed a BF_10_ of 1549.23. These two values are considered extreme evidence for the research hypothesis regarding the numerical tasks (Beard et al., 2016). The strong support for the slower performance of the MLD group compare to controls was not found for the non-numerical tasks, and the analyses revealed a BF_10_ of 2.77 and 0.37 for the shape comparison task and semantic judgment task, respectively.

Bayesian statistical analyses were also conducted in order to provide further support for the data, using JASP statistical software 0.9.2 with the default prior values (JASP, 2019; Rouder et al., 2012). Analyzing the significant difference between the two groups in their performance of the numeric comparison task revealed a BF_10_ of 15.76. This value is considered strong evidence for the research hypothesis (Beard et al., 2016) and suggests that given the data, it is 15.76 times more likely that MLD have higher SD than controls when performing the numeric comparison task, than the alternative. The same pattern of results was found for the equations judgment task and the analysis revealed a BF_10_ of 244.71, which is considered extreme evidence for the research hypothesis. This strong support for the higher SD among MLD compared to controls when performing the numerical tasks was not found for the non-numerical tasks. The analyses revealed a BF_10_ of 0.68 and 0.34 for the shape comparison task and semantic judgment task, respectively.

Bayesian statistical analyses were also conducted in order to provide further support for the data, using JASP statistical software 0.9.2 with the default prior values (JASP, 2019; Rouder et al., 2012). Analyzing the significant difference between the two groups in their performance of the numeric comparison task revealed a BF_10_ of 15.77. This value is considered strong evidence for the research hypothesis (Beard et al., 2016) and suggests that given the data, it is 15.77 times more likely that MLD have higher mu’s compared to controls when performing the numeric comparison task, than the alternative. The same pattern of results was found for the equations judgment task, and the analysis revealed a BF_10_ of 290.27, which is considered extreme evidence for the research hypothesis. This strong support for the higher mu’s among MLD compared to controls when performing the numerical tasks, was not found for the non-numerical tasks. The analyses revealed a BF_10_ of 3.89 and 0.40 for the shape comparison task and semantic judgment task, respectively. While the BF_10_ value of the shape comparison analysis is considered moderate evidence for the research hypothesis, altogether the analyses revealed higher support for the differences between the two groups in the numerical tasks than in the non-numerical tasks.

Bayesian statistical analyses were also conducted in order to provide further support for the data, using JASP statistical software 0.9.2 with the default prior values (JASP, 2019; Rouder et al., 2012). Analyzing the significant difference between the two groups in their performance of the numeric comparison task revealed a BF_10_ of 5.37. This value is considered moderate evidence for the research hypothesis (Beard et al., 2016) and suggests that, given the data, it is 5.37 times more likely that MLD will have higher sigma’s compared to controls when performing the numeric comparison task, than the alternative. The same pattern of results was found for the equations judgment task and the analysis revealed a BF_10_ of 19.28, which is considered strong evidence for the research hypothesis. This strong support for the higher sigma’s among MLD compared to controls when performing the numerical tasks was not found for the non-numerical tasks. The analyses revealed a BF_10_ of 1.31 and 0.38 for the shape comparison task and semantic judgment task, respectively.

Bayesian statistical analyses were also conducted in order to provide further support for the data, using JASP statistical software 0.9.2 with the default prior values (JASP, 2019; Rouder et al., 2012). Analyzing the significant difference between the two groups in their performance of the numeric comparison task revealed a BF_10_ of 62.23. This value is considered very strong evidence for the research hypothesis (Beard et al., 2016) and suggests that, given the data, it is 62.23 times more likely that MLD will have higher tau’s compared to controls when performing the numeric comparison task, than the alternative. The same pattern of results was found for the equations judgment task, and the analysis revealed a BF_10_ of 704.96, which is considered extreme evidence for the research hypothesis. This strong support for higher tau’s among MLD compared to controls when performing the numerical tasks, was not found for the non-numerical tasks. The analyses revealed a BF_10_ of 0.53 and 0.34 for the shape comparison task and semantic judgment task, respectively.
